# Intensification of constructed wetlands for land area reduction: a review

**DOI:** 10.1007/s11356-017-8740-z

**Published:** 2017-03-11

**Authors:** Huma Ilyas, Ilyas Masih

**Affiliations:** 1 Kievitlaan 9, 2289ED Rijswijk, The Netherlands; 2 UNESCO-IHE Institute for Water Education, Westvest 7, 2611AX Delft, The Netherlands

**Keywords:** Constructed wetland, Dissolved oxygen, Land area, Nitrogen, Organic matter, Phosphorus, Wastewater

## Abstract

The large land area requirement of constructed wetlands (CWs) is a major limitation of its application especially in densely populated and mountainous areas. This review paper provides insights on different strategies applied for the reduction of land area including stack design and intensification of CWs with different aeration methods. The impacts of different aeration methods on the performance and land area reduction were extensively and critically evaluated for nine wetland systems under three aeration strategies such as tidal flow (TF), effluent recirculation (ER), and artificial aeration (AA) applied on three types of CWs including vertical flow constructed wetland (VFCW), horizontal flow constructed wetland (HFCW), and hybrid constructed wetland (HCW). The area reduction and pollutant removal efficiency showed substantial variation among different types of CWs and aeration strategies. The ER-VFCW designated the smallest footprint of 1.1 ± 0.5 m^2^ PE^−1^ (population equivalent) followed by TF-VFCW with the footprint of 2.1 ± 1.8 m^2^ PE^−1^, and the large footprint was of AA-HFCW (7.8 ± 4.7 m^2^ PE^−1^). When footprint and removal efficiency both are the major indicators for the selection of wetland type, the best options for practical application could be TF-VFCW, ER-HCW, and AA-HCW. The data and results outlined in this review could be instructive for futures studies and practical applications of CWs for wastewater treatment, especially in land-limited regions.

## Introduction

Constructed wetlands (CWs) are environmentally friendly and cost-effective option to treat wastewater. In CWs, the interaction of plants, microorganisms, and soil leads to natural processes (physical, chemical, and biological), which are used to remove pollutants from wastewater (Vymazal 2005). Two designs of CWs are generally used, which are free water surface flow constructed wetland (FWSCW) and subsurface flow constructed wetland (SSFCW). Among the SSFCW, two types exist, which are horizontal flow constructed wetland (HFCW) and vertical flow constructed wetland (VFCW). Due to the limited treatment performance and different pollutant removal mechanisms of all types of CWs, the hybrid constructed wetland (HCW), the combination of VFCW and HFCW, one next to the other has been developed, for the purpose of mainly nitrification-denitrification treatment trains to achieve better total nitrogen (TN) removal (Cooper et al. 1999; Kadlec and Wallace 2009).

Although much development has taken place within the CWs to enhance the efficiency of the system, some limitations of all types of CWs still remain. The major limitations are poor nutrient (nitrogen and phosphorus) removal, large land area requirements (referred in this document as footprint), and oxygen transfer limitation. The design of CWs depends on many factors such as required effluent quality, footprint, technology combinations, and use of energy. The other favorable considerations for CWs are that they are simple in construction as well as in operation and maintenance, have high robustness and process stability, high buffer capacity for hydraulic and organic load fluctuations, and a low sludge production (Langergraber et al. 2010). Several research studies has been dedicated to explore possibility of reducing footprint besides achieving good quality effluent (Kantawanichkul et al. 2003; Ye and Li 2009; Zhang et al. 2010; Prost-Boucle and Molle 2012; Foladori et al. 2012, 2013; Zapater-Pereyra et al. 2014, 2015).

The small footprint is most needed in the mountainous and densely populated regions, where land resources are scarce and land costs are high. The land availability and cost may limit the applicability of the system because CW treatment may be economically feasible option only where land is available at low cost. To overcome this limitation, some researchers have suggested to increase the depth of the system without increasing the footprint, the stack design of different stages to reduce the footprint. In addition to that, an extensive research has indicated that if sufficient oxygen within the system is available, it gives microorganisms the conditions to complete biodegradation and enhances the system efficiency for organic matter and nitrogen removal and, thus, reduce the system footprint.

The knowledge published in international journals and books on enhanced treatment performance of intensified CWs has substantially increased in recent years. Zhang et al. (2014) indicated that the removal of total suspended solids (TSSs) in HFCW and VFCW was <80 and >85%, respectively, the removal of biochemical oxygen demand (BOD) in HFCW and VFCW was >75 and 90%, respectively, whereas chemical oxygen demand (COD) removal in HFCW and VFCWs was >65%. Nevertheless, HCWs were proved to be more effective for the removal of TSS, BOD, and COD up to >90, >85, and >85%, respectively. A review on the performance of CWs for nutrients removal conducted by Vymazal (2007) documented that the removal of TN varied between 40 and 55% with removed load ranging between 250 and 630 g N m^−2^ yr^−1^, and the removal of total phosphorus (TP) varied between 40 and 60% with removed load ranging between 45 and 75 g P m^−2^ yr^−1^ depending on CW type and inflow loading. The removal of nitrogen and phosphorus via harvesting of aboveground biomass of emergent vegetation is low (100–200 g N m^−2^ yr^−1^, 10–20 g P m^−2^ yr^−1^, respectively), but for lightly loaded systems, it could be significant. Many research studies have indicated that substrate of high adsorption capacity enhances the removal of phosphorus in CWs. The enhanced performance of CWs using different substrates for phosphorus removal was reviewed in Vohla et al. (2011). Despite the general viewpoint that phosphorus removal is mainly by adsorption on substrate media, precipitation, and uptake by the plants, an increased dissolved oxygen (DO) level might accelerate phosphorus adsorption and precipitation to the substrate.

Since the last few decades, the focus of the research was on how to reduce the footprint of CWs by stacking up different stages and to intensify them to overcome the constraints such as large footprint and oxygen transfer limitation, which hindered the low cost and high treatment performance. Despite growing number of studies, a comprehensive review on the performance of these stacked up and intensified systems is lacking while considering the reduction in footprint. This review is an original and novel attempt that examined the available evidence on the effects of stacking up and aeration methods in different types of CWs on footprint reduction. The main objective of this review paper is to provide a critical and comprehensive evaluation of the intensified CWs for their footprint and treatment efficiency. The synthesis presented in this paper attempts to aid in better understanding of this current technology for best possible design to achieve reduction in footprint and highlight innovative ideas for intensification to improve the treatment efficiency. Therefore, it is necessary to review and discuss the recent developments on design of CWs for the reduction in footprint and intensification of CWs with different aeration methods. The optimization of DO in VFCW, HFCW, and HCW with different strategies such as tidal flow (TF), effluent recirculation (ER), and artificial aeration (AA) as well as the approaches for reducing the footprint of CWs are summarized in this paper. Another significant and novel feature of this research is that it has accumulated and done additional calculations, when necessary, for a number of parameters, which are important for design and treatment process understanding. A detailed comparative and critical analysis of the performance and contributing factors and processes is presented in this work. The information outlined in this manuscript could be instructive for future studies and practical applications for wastewater treatment, especially when footprint and treatment efficiency are the major consideration.

## Methodology

Research articles, research papers, and reviewed papers and books were searched from several sources, such as Google Scholar, Scopus, and individual journal websites, related to the different strategies used for the footprint reduction of CWs including design and performance enhancement of the intensified CWs for organic matter and nutrient removal while reducing the footprint. The search resulted in accumulation of about 100 documents, which were further selected to meet the purpose of this research. Considering the objective of this review paper, the design and treatment performance of nine different types of CWs were analyzed that include VFCW, HFCW, and HCW with TF, ER, and AA. Different parameters such as wastewater type, depth, area, hydraulic loading rate (HLR), organic loading rate (OLR), DO, fill and drain time ratio, recirculation flow ratio (RFR), airflow rate (AFR), TSS, COD, ammonium-nitrogen (NH_4_^+^-N), TN, and TP were considered for the comparison of different aeration strategies. These parameters were gathered from the reviewed studies or estimated using the information available in those studies. Furthermore, statistical analysis was carried out and descriptive statistics were computed for some indicators where adequate data were available.

## Results and discussion

In this section, the focus is on the footprint and oxygen transfer limitation of CWs, which are the main subjects of this study. The footprint and different strategies for its reduction are presented in detail.

### Footprint (land area requirement)

The research have shown that the CWs may require large footprint to assure a good quality effluent (Kivaisi 2001; Foladori et al. 2013). The footprint could be even higher when different CWs are combined to form a HCW (Foladori et al. 2012). However, the footprint of CWs varies in different countries (Table 1). The Environmental Protection Agency (EPA) design recommends reducing the footprint to 1 m^2^ PE^−1^ (PE refers to population equivalent) for tertiary treatment and 0.5 m^2^ PE^−1^ for storm water treatment. However, some operators recommend 10 and 5 m^2^ PE^−1^ for secondary and tertiary treatments, respectively. In Europe, VFCWs are generally sized 1–3 m^2^ PE^−1^ and HFCWs are designed around 5 m^2^ PE^−1^ (Vymazal 2011) for the removal of organics and TSS, but this area may not be sufficient for the removal of nutrients (Babatunde et al. 2008). Some studies even suggested very large area requirement of 15–30 and 40–70 m^2^ PE^−1^, for the removal of nitrogen (<8 mg L^−1^) and phosphorus (<1.5 mg L^−1^), respectively (Schierup et al. 1990, cited in Babatunde et al. 2008).

**Table 1:** The recommended footprint of CWs in different countries

CW type	Area (m^2^ PE^−1^)	Regions	Author
VFCW	3.2	Denmark	Brix and Arias (2005)
	1–3	Europe	Vymazal (2011)
HFCW	5	Europe	Vymazal (2011)
HCW	5	Czech Republic	Vymazal (1996)
	5	Ireland	Babatunde et al. (2008)
	0.6–1.2	China (South)	Zhang et al. (2009)
	3–21	China (North)	Zhang et al. (2009)

The population equivalent (PE) is calculated based on the common relation 1 PE = 60 g BOD d^−1^

*CW* constructed wetland, *VFCW* vertical flow constructed wetland, *HFCW* horizontal flow constructed wetland, *HCW* hybrid constructed wetland

Additionally, the footprint differs between climatic regions (US EPA 2000). For instance, in China, the southern China belongs to the subtropical climate, with relatively high temperature and a humid climate, which is most favorable for wetland vegetation. So, the footprint for CWs is much smaller in the southern than the northern regions in China (Table 1) (Li and Wang 2006, cited in Zhang et al. 2009). However, in southern China, the average population density is 210 persons km^−2^. Due to high population density, land resources are scarce and land costs are high. Therefore, CW treatment technology may be cost-effective option only, where land is abundant and available at low cost (Zhai et al. 2006). To overcome this limitation, some researchers suggest increasing the CW depth without increasing the footprint, the principle of stacking up different stages to reduce the footprint.

#### Stacking up different stages to reduce the footprint

Some studies use the perception of increasing the system depth instead of increasing the footprint (Table 2).

**Table 2:** Comparison of studies using stack design of CWs to reduce the footprint

Wetland type/scale	Depth (m)	Area (m^2^ PE^−1^)	HLR (m^3^ m^−2^ d^−1^)	OLR (g COD m^−2^d^−1^)	TSS (Rem %)	COD (Rem %)	NH_4_^+^-N (Rem %)	TN (Rem %)	TP (Rem %)	Author
HCW										
Pilot ^(V + H)^	1.4	4.7	0.03	32	98	91	84	76	97	Kantawanichkul et al. (2003)
Pilot ^(V + H)^	1.4	2.6	0.06	71	99	86	66	57	93	Kantawanichkul et al. (2003)
Pilot ^(V + H)^	1.4	1.8	0.12	137	99	86	85	75	55	Kantawanichkul et al. (2003)
Pilot ^(H + V)^	0.6/0.6	4.2	0.03	37	99	95	98	79	99	Kantawanichkul et al. (2003)
Pilot ^(H + V)^	0.6/0.6	1.9	0.06	70	99	86	86	64	90	Kantawanichkul et al. (2003)
Pilot ^(H + V)^	0.6/0.6	1.5	0.12	147	99	79	87	73	63	Kantawanichkul et al. (2003)
Pilot ^(H + F + H)^	1.0	2.3	0.16	51	87	85	81	82	67	Ye and Li (2009)
Pilot ^(H + F + H)^	1.0	1.2	0.32	102	89	85	83	83	64	Ye and Li (2009)
Pilot ^(V)^	0.6	3.2	0.06	37	NA	82	70	24	49	Foladori et al. (2012)
Pilot ^(V + H)^	0.6/0.6	6.4	0.03	18	NA	94	80	78	98	Foladori et al. (2012)
Pilot ^(V)^	0.6	1.3	0.12	87	NA	74	59	40	36	Foladori et al. (2012)
Pilot ^(V + H)^	0.6/0.6	2.6	0.06	43	NA	88	69	75	64	Foladori et al. (2012)
Lab ^(V + H)^	0.8/0.35	7.9	0.046	15	91	87	85	72	80	Zapater-Pereyra et al. (2015)
Lab ^(V + H)^	0.8/0.35	3.4	0.046	27	93	93	73	82	61	Zapater-Pereyra et al. (2015)
Lab ^(V + H)^	0.8/0.35	2.6	0.046	37	84	91	55	78	44	Zapater-Pereyra et al. (2015)

The population equivalent (PE) is calculated based on the common relation 1 PE = 60 g BOD d^−1^. Biochemical oxygen demand (BOD) values were approximated using the ratio COD/BOD = 2 in the studies where BOD was not reported (Ye and Li 2009; Foladori et al. 2012; Zapater-Pereyra et al. 2015)

*CW* constructed wetland, *HCW* hybrid constructed wetland, *HLR* hydraulic loading rate, *OLR* organic loading rate, *TSSs* total suspended solids, *COD* chemical oxygen demand, *NH*_*4*_^*+*^ *-N* ammonium-nitrogen, *TN* total nitrogen, *TP* total phosphorus, *Rem* removal, *NA* not available, *VFCW* vertical flow constructed wetland over horizontal flow sand bed (^V + H^), *H + V* HFCW horizontal flow constructed wetland and VFCW connected in series, *H + F + H HFCW* free water surface flow CW and HFCW in a stack design, *V VFCW*

##### Case studies on HCWs

For the comparative studies of two different designs of HCW, Kantawanichkul et al. (2003) designed two pilot-scale HCWs for the treatment of pig farm wastewater. In one HCW, the VFCW was over the horizontal flow sand bed, and in the other HCW, the HFCW and VFCW were connected in series. The performance of the systems was tested at different HLRs. The result showed that the wastewater treatment performance of both types of HCWs was similar and the removal of TP decreased due to the limited adsorption capacity of sand media (Table 2). However, the system with a VFCW over horizontal flow sand bed is more suitable for sites with limited land area as it can reduce the footprint to 1.8 m^2^ PE^−1^ (Table 2).

Ye and Li (2009) designed the towery HCW consisting of three stages; the first and third stages were rectangle HFCWs, and the second stage was a circular three-layer FWSCW. Although the purpose of towery HCW was to enhance the removal of nitrogen, it also used the principle of stacking up different stages to reduce the footprint up to 1.2 m^2^ PE^−1^. Nitrification rates were enhanced by passive aeration of a tower overflow from the upper layer into the lower layer in the second stage of the wetland and denitrification rates by additional organic matter supplied as a result of bypass influent directly into the second stage (Table 2).

Foladori et al. (2012) proposed a novel design of HCW consisted of vertical flow filter (VFF) and HFCW. The wastewater entered the VFF and the effluent of VFF drained and flowed by gravity into the HFCW since VFF was on top of the HFCW. The HCW was tested for low and high OLRs. The VFF contributed more in the treatment of COD and NH_4_^+^-N than the HFCW, whereas HFCW played major role in the removal of TN. For instance, the total removal of COD by HCW was 94%, and 82% were removed by VFF. Similarly, total removal of NH_4_^+^-N was 80%, and 70% were removed by VFF. The total removal of TN was 78%, and 54% were removed by HFCW. The footprint of the VFF and HFCW was 1.3 and 2.6 m^2^ PE^−1^, respectively, which is not common in the design of the VFCW and HFCW (Table 2).

Zapater-Pereyra et al. (2015) designed the Duplex-CW, a hybrid system with a stacked arrangement of a VFCW on top of a horizontal flow filter (HFF). The fill and drain Duplex-CW was tested with three different strengths of wastewater (low, medium, and high). The performance of fill and drain was good even for the treatment of high-strength wastewater. The VFCW contributed more in treatment than the HFF. The footprint of the system was reduced from 7.9 to 2.6 m^2^ PE^−1^, which is two to three times lower area than a single VFCW to reach similar TN effluent concentrations (Table 2).

### Strategies to combat oxygen transfer limitation

The oxygen transfer in conventional HFCW is poor and inconsistent, and it occurs mainly through convection and diffusion from the air to the surface water with estimated oxygen transfer rates of 0.3–3.2 g O_2_ m^−2^ d^−1^ (Tyroller et al. 2010). In conventional VFCW, the oxygen transfer is through intermittent loading; thus, these systems achieve oxygen transfer rates of 28–100 g O_2_ m^−2^ d^−1^ (Cooper et al. 1999). It has been reported that DO above 1.50 mg L^−1^ is essential for nitrification, whereas denitrification occurs below 0.50 mg L^−1^ (Ye and Li 2009).

To overcome the oxygen transfer limitation, different aeration strategies are applied on CWs such as TF, ER, and AA.

#### Tidal flow (TF)

This operation strategy expected to improve the removal of organic matter and nutrients in CWs because it involves the filling and draining of the wastewater in the bed, which increases the entrance of fresh air into the system. This technology has been demonstrated in multiple studies (Table 3). The TF system can provide advanced, biological nitrogen removal with less energy than activated sludge system and requires smaller footprint than conventional CWs for wastewater treatment. This system also needs about half of the power compared with aerated wetlands (Austin and Nivala 2009).

**Table 3:** Comparison of studies using TF

Wetland type/scale	WT	Depth (m)	Area (m^2^ PE^−1^)	HLR (m^3^ m^−2^ d^−1^)	OLR (g COD m^−2^ d^−1^)	OM	Fill and drain time ratio (h:h)	Effluent DO (mg L^−1^)	TSS (Rem %)	COD (Rem %)	NH_4_^+^-N (Rem %)	TN (Rem %)	TP (Rem %)	Author
VFCW														
Pilot	P	0.6	0.25	0.12	330	IF	NA	NA	78	80	58	NA	NA	1
Pilot	P	1.0	0.25	0.12	330	IF	NA	NA	78	80	58	NA	NA	1
Lab	S	0.65	3.3	0.1	36	IF	1:2	6.96	NA	96	94	47	91.9	2
Lab	S	0.65	3.3	0.1	36	IF	2:1	6.87	NA	97	90	56	92.2	2
Lab	S	0.65	3.3	0.1	36	CF	3:0	5.28	NA	92	63	67	87.5	2
Lab^1^	S	0.65	4.0	0.08	30	IF	1:2	7.0	NA	96	94	47	NA	3
Lab^1^	S	0.65	4.0	0.08	30	CF	3:0	5.3	NA	92	63	67	NA	3
Lab^2^	S	0.65	4.0	0.08	30	IF	4:3	7.5	NA	93	76	67	NA	3
Lab^2^	S	0.65	4.0	0.08	30	CF	7:0	6.8	NA	94	78	69	NA	3
Pilot	AF	1.1	5.0	0.29	118	IF	4:4	NA	83	84	93	78	94	4
Pilot	AF	1.1	0.3	0.29	376	IF	4:4	NA	46	36	49	11	75	4
Lab	S	1.5	0.3	0.90	345	IF	3:3	3.2	NA	NA	82	43	NA	5
Lab	S	1.5	0.2	0.90	650	IF	3:3	2.3	NA	NA	33	21	NA	5
Lab ^D^/^U^	P	0.7	2.2	0.44	88	IF	1:1	2.0–4.7	86	70	96	60	88	6
Lab ^D^/^U^	P	0.7	0.6	0.44	264	IF	1:1	1.0	85	62	94	60	88	6
Pilot	S	1.8	1.0	0.39	114	IF	3:3	2.8	NA	93	93.3	74.5	NA	7
Pilot	S	1.8	0.3	0.39	436	IF	3:3	2.8	NA	93	93.3	74.5	NA	7
HFCW														
Pilot	S	0.6	14	0.03	8.4	IF	NA	NA	NA	96	95	NA	67	8
Pilot	S	0.6	14	0.03	8.4	CF	NA	NA	NA	95	81	NA	43	8
HCW														
Lab^(V + H)^	D	0.8/0.35	7.9	0.046	15	IF	1:2	2.5	91	87	85	72	80	9
Lab^(V + H)^	D	0.8/0.35	3.4	0.046	27	IF	1:2	2.5	93	93	73	82	61	9
Lab^(V + H)^	D	0.8/0.35	2.6	0.046	37	IF	1:2	2.5	84	91	55	78	44	9

Fill and drain time ratio is given in days (Jia et al. 2010, 2011; Zapater-Pereyra et al. 2015). The population equivalent (PE) is calculated based on the common relation 1 PE = 60 g BOD d^−1^. Biochemical oxygen demand (BOD) values were approximated using the ratio COD/BOD = 2 in the studies where BOD was not reported (Jia et al. 2010, 2011; Zhang et al. 2012; S. Wu et al. 2015a; Zapater-Pereyra et al. 2015). (1) Sun et al. (2006), (2) Jia et al. (2010), (3) Jia et al. (2011), (4) Zhao et al. (2011), (5) Wu et al. (2011), (6) Hu et al. (2014), (7) S. Wu et al. (2015a), (8) Zhang et al. (2012), (9) Zapater-Pereyra et al. (2015)

*TF* tidal flow, *VFCW* vertical flow constructed wetland, *HFCW* horizontal flow constructed wetland, *HCW* hybrid constructed wetland, *WT* wastewater type, *P* piggery, *S* synthetic, *AF* animal farm, *D* domestic, *HLR* hydraulic loading rate, *OLR* organic loading rate, *OM* operation mode, *IF* intermittent flood, *CF* continuous flood, *h* hour, *DO* dissolved oxygen, *TSSs* total suspended solids, *COD* chemical oxygen demand, *NH*_*4*_^*+*^ *-N* ammonium-nitrogen, *TN* total nitrogen, *TP* total phosphorus, *NA* not available, *1* VFCW, *2* free water surface flow constructed wetland (FWSCW), *D* VFCW downflow, *U* VFCW upflow, *V + H* VFCW over horizontal flow filter

##### Influence of TF on footprint reduction

Various studies clearly demonstrated that in fill and drain system, the sufficient DO enhanced the treatment efficiency and the footprint of the TF-VFCW was reduced to 0.3–5.0 m^2^ PE^−1^(Table 3). In TF-HCW, Zapater-Pereyra et al. (2015) achieved that fill and drain performed well even to treat high-strength wastewater (Table 3), but the contribution of both compartments (VFCW and HFF) was different. The contribution of VFCW was more for COD, NH_4_^+^-N, and TP removal, but the HFF contributed more for TN removal. For instance, the total removal of COD by HCW was 93%, and the VFCW contributed for 65% removal and the rest of the 28% was removed by HFF (Table 3). The removal of NH_4_^+^-N in HCW with low-, medium-, and high-strength wastewater was 85, 73, and 55%, respectively, and VFCW contributed for the removal of 82, 51, and 46%, respectively. Similarly, the total removal of TP was 61% and the VFCW contributed for the 50% removal and the rest of the 11% was removed by HFF (Table 3). Whereas with low-, medium-, and high-strength wastewater, the removal of TN in HCW was 72, 82, and 78%, respectively, and HFF contributed for the removal of 38, 49, and 48%, respectively (Table 3). The footprint of the system was reduced to 2.6 m^2^ PE^−1^, which is two to three times lower area than a single VFCW to reach similar TN effluent concentrations (Table 3).

Limited evidence suggested that the TF-HFCW required large footprint (14 m^2^ PE^−1^) than TF-VFCW (0.3–0.5 m^2^ PE^−1^) and TF-HCW (2.6–7.9 m^2^ PE^−1^) (Table 3). The influence of TF in VFCW, HFCW, and HCW for the removal of organic matter, nitrogen, and phosphorus to compare the contribution in footprint reduction by some other studies can be seen in Table 3.

#### Effluent recirculation (ER)

The perception behind the ER is to increase the aerobic microbial activity through the excessive interaction between pollutants and microorganism without substantial alterations in the approach. The ER has been proposed by many researchers (Table 4) as an operational modification to improve the effluent quality of CWs, and in ER, a part of effluent is extracted and transferred back to the inflow of the system. In HFCW and VFCW, the ER with a ratio of 0.5 to 2.5 was mostly applied (Wu et al. 2014).

**Table 4:** Comparison of studies using ER

Wetland type/scale	WT	Depth (m)	Area (m^2^ PE^−1^)	HLR (m^3^ m^−2^ d^−1^)	OLR (g COD m^−2^ d^−1^)	RFR (R vol:I vol)	TSS (Rem %)	COD (Rem %)	NH_4_^+^-N (Rem %)	TN (Rem %)	TP (Rem %)	Author
VFCW												
Pilot	P	1.0	1.6	0.06	86	NA	91.2	77.6	70.4	NA	NA	1
Pilot	P	1.0	1.0	0.08	133	0.5:1	49 to 77	NA	36 to 44	NA	42 to 49	2
Pilot	P	1.0	0.5	0.15	265	0.5:1	49 to 77	NA	36 to 44	NA	42 to 49	2
Pilot	D	0.6	0.5	0.5	270	NA	90	84	92	NA	NA	3
Full	D	0.8	1.1	0.4	350	0.5:1	90	83	53	NA	NA	4
Full	D	0.8	1.6	0.4	300	1:1	95	90	58	NA	NA	4
Pilot	D	0.6	1.4	0.17	83	0.6:1	73 to 76	80 to 84	79 to 72	29 to 44	29 to 21	5
HFCW												
Pilot	S	1.0	12	0.01	8.0	0.5:1	NA	88 to 85	79 to 38	NA	77 to 65	6
Pilot	S	1.0	6.0	0.03	15	0.5:1	NA	88 to 85	79 to 38	NA	77 to 65	6
HCW												
Pilot ^(V + H)^	P	1.2	1.1	0.04	105	0.5:1	NA	97	99	85	NA	7
Pilot ^(V + H)^	P	1.4	4.7	0.03	32	1:1	98	91	84	76	97	8
Pilot ^(V + H)^	P	1.4	2.6	0.06	71	1:1	99	86	66	57	93	8
Pilot ^(V + H)^	P	1.4	1.8	0.12	137	1:1	99	86	85	75	55	8
Pilot ^(H + V)^	P	0.6/0.6	4.2	0.03	37	1:1	99	95	98	79	99	8
Pilot ^(H + V)^	P	0.6/0.6	1.9	0.06	70	1:1	99	86	86	64	90	8
Pilot ^(H + V)^	P	0.6/0.6	1.5	0.12	147	1:1	99	79	87	73	63	8
Lab ^(V + H)^	P	1.0	3.6	0.08	83	1:1	92	58	50	50	50	9
Pilot ^(V + V)^	O	0.6/0.4	1.8	0.04	110	1:1	87 to 97	75 to 94	NA	73	59 to 73	10
Pilot ^(H + V)^	D	0.8/0.8	17	0.06	14	1:1	NA	NA	NA	79	NA	11
Pilot ^(H + V)^	D	0.8/0.8	5.5	0.13	37	1:1	NA	NA	NA	79	NA	11
Full ^(V + V + V + H + V)^	P	0.8	1.7	0.007	53	2.6:1	87 to 98	72 to 93	57 to 88	50 to 71	75 to 92	12

The population equivalent (PE) is calculated based on the common relation 1 PE = 60 g BOD d^−1^. Biochemical oxygen demand (BOD) values were approximated using the ratio COD/BOD = 2 in the studies where BOD was not reported (Kantawanichkul et al. 2001; Kantawanichkul and Somprasert 2005). (1) Sun et al. (2003), (2) Lian-sheng et al. (2006), (3) Sklarz et al. (2009), (4) Prost-Boucle and Molle (2012), (5) Foladori et al. (2013), (6) Stefanakis and Tsihrintzis (2009), (7) Kantawanichkul et al. (2001), (8) Kantawanichkul et al. (2003), (9) Kantawanichkul and Somprasert (2005), (10) Travis et al. (2012), (11) Ayaz et al. (2012), (12) Zhang et al. (2016)

*ER* effluent recirculation; *VFCW* vertical flow constructed wetland; *HFCW* horizontal flow constructed wetland; *HCW* hybrid constructed wetland; *WT* wastewater type; *P* piggery; *D* domestic; *S* synthetic; *O* oil-rich; *HLR* hydraulic loading rate; *OLR* organic loading rate; *RFR* recirculation flow ratio, recirculated volume to influent volume; *TSSs* total suspended solids; *COD* chemical oxygen demand; *NH*_*4*_^*+*^ *-N* ammonium-nitrogen; *TN* total nitrogen; *TP* total phosphorus; *NA* not available; *V + H* VFCW over horizontal flow sand bed; *H + V* HFCW and VFCW connected in series; *V + V* VFCW and VFCW connected in series; *V + V + V + H + V* four VFCW and one HFCW connected in series

##### Influence of ER on footprint reduction

The footprint of different types of CWs is different with the application of ER. In ER-VFCW, the footprint was reduced to 0.5–1.6 m^2^ PE^−1^ (Table 4). For instance, Prost-Boucle and Molle (2012) investigated the use of ER on a full-scale single French VFCW for the treatment of domestic wastewater to replace the classical French VFCW, which comprises two stages of treatment. This single-stage VFCW with a smaller footprint of 1.1 to 1.6 m^2^ PE^−1^ had the similar treatment performance as the classical French system with two successive stages of 2 m^2^ PE^−1^ studied by Troesch et al. (2010). It was concluded that ER has enhanced the performance of CWs for wastewater treatment, which is central in the reduction of footprint (Table 4).

Foladori et al. (2013) studied the application of ER on pilot-scale VFCW for the treatment of domestic wastewater. The establishment of simultaneous nitrification and denitrification conditions facilitated the high removal of TN with the value of 6.1 g N m^−2^ d^−1^, whereas the conventional VFCW could remove only 1.5 g N m^−2^ d^−1^. The footprint of the ER-VFCW was reduced to 1.4 m^2^ PE^−1^ from the footprint 3.6 m^2^ PE^−1^ of the conventional downflow VFCW (Table 4).

Similarly, in ER-HCW, the reduction in footprint was achieved. For instance, Kantawanichkul et al. (2003) designed two pilot-scale HCWs for the comparative studies of two different designs of HCW to investigate the effect of ER on the treatment performance of the systems. In one HCW, the VFCW was over the horizontal flow sand bed, and in the other HCW, the HFCW and VFCW were connected in series. The treatment performance of the systems was good, which resulted in the reduction of footprint to 1.8 and 1.5 m^2^ PE^−1^, respectively (Table 4). Moreover, based on the availability of limited number of data points, it is suggested that the ER-HFCW required large footprint (6–12 m^2^ PE^−1^) than ER-VFCW (0.5–1.6 m^2^ PE^−1^) and ER-HCW (1.5–4.7 m^2^ PE^−1^) (Table 4).

The impact of ER in VFCW, HFCW, and HCW for the removal of organic matter, nitrogen, and phosphorus and comparison of this strategy to contribute in footprint reduction by multiple studies can be seen in Table 4.

#### Artificial aeration (AA)

The AA has been proposed as a solution to create an aerobic condition promising for nitrification to improve the performance of all types of CWs. However, the use of AA with air pump and air blower is mostly in VFCW and HFCW. The efficacy of the approach has been shown at laboratory scale and pilot scale in VFCW and HFCW (Tables 5 and 6).

**Table 5:** Comparison of studies using AA in VFCW

Wetland type/scale	WT	Depth (m)	Area (m^2^ PE^−1^)	HLR (m^3^ m^−2^ d^−1^)	OLR (g COD m^−2^ d^−1^)	AM	AP	AFR (m^3^ h^−1^)	Effluent DO (mg L^−1^)	TSS (Rem %)	COD (Rem %)	NH_4_^+^-N (Rem %)	TN (Rem %)	TP (Rem %)	Author
VFCW															
Lab	D	0.7	5.7	0.2	48	IA	B	0.25	1.0	90 to 96	76 to 81	78 to 87	65 to 70	74 to 74	1
Lab	R	0.8	9.9	0.19	12	CA	M	NA	4.4	NA	65 to 81	61 to 87	38 to 48	31 to 37	2
Lab	R	0.8	9.9	0.19	12	IA	M	NA	3.0	NA	65 to 78	61 to 78	38 to 57	31 to 35	2
Lab	S	0.6	5.2	0.002	23	CA	M	1.18	4.06	NA	86	78	69	NA	3
Lab	S	0.6	5.2	0.002	23	IA	M	1.18	2.65	NA	82	77	70	NA	3
Lab	S	0.65	1.6	0.21	73	CA	B	0.09	7.0	NA	97	99	29	NA	4
Lab	S	0.65	1.6	0.21	73	IA	B	0.09	0.5–7.0	NA	96	97	74	NA	4
Lab	S	0.65	4.0	0.07	30	IA	B	0.09	8.01	NA	75 to 96	25 to 99	26 to 90	55 to 91	5
Pilot	D	0.6	1.8	0.16	64	IA	B	3.5	NA	73 to 86	80 to 88	79 to 66	29 to 49	29 to 24	6
Pilot	D	0.85	2.2	0.095	54	CA	B	2.2	8.1	NA	NA	98	58	NA	7
Pilot	D	0.85	2.2	0.095	54	IA	B	2.2	6.3	NA	NA	89	78	NA	7
Lab	S	0.65	9.7	0.01	12	IA	B	1.86	4.06	NA	63 to 97	21 to 99	27 to 90	52 to 91	8
Lab	S	0.5	NA	NA	NA	CA	S	0.0004	0.41–2.82	NA	61 to 74	52 to 77	54 to 76	66 to 70	9
Lab	S	0.5	NA	NA	NA	CA	M	0.0004	1.23–2.32	NA	61 to 81	52 to 83	54 to 67	66 to 69	9
Lab	S	0.5	NA	NA	NA	CA	B	0.0004	0.42–1.85	NA	61 to 75	52 to 75	54 to 67	66 to 70	9
Lab	D	0.65	1.4	0.2	85	IA	B	0.06	6.0–8.0	NA	97	98	91	NA	10

The population equivalent (PE) is calculated based on the common relation 1 PE = 60 g BOD d^−1^. Biochemical oxygen demand (BOD) values were approximated using the ratio COD/BOD = 2 in the studies where BOD was not reported (Liu et al. 2013; Fan et al. 2013a, b; H. Wu et al. 2015b, 2016a). (1) Tao et al. (2010), (2) Dong et al. (2012), (3) Liu et al. (2013), (4) Fan et al. (2013a), (5) Fan et al. (2013b), (6) Foladori et al. (2013), (7) Boog et al. (2014), (8) H. Wu et al. (2015b), (9) Wang et al. (2015), (10) H. Wu et al. (2016a)

*AA* artificial aeration, *VFCW* vertical flow constructed wetland, *WT* wastewater type, *D* domestic, *R* river, *S* synthetic, *HLR* hydraulic loading rate, *OLR* organic loading rate, *AM* aeration mode, *CA* continuous aeration, *IA* intermittent aeration, *AP* aeration position, *B* bottom, *M* middle, *S* surface, *AFR* airflow rate, *DO* dissolved oxygen, *TSSs* total suspended solids, *COD* chemical oxygen demand, *NH*_*4*_^*+*^ *-N* ammonium-nitrogen, *TN* total nitrogen, *TP* total phosphorus, *NA* not available

**Table 6:** Comparison of studies using AA in HFCW and HCW

Wetland type/scale	WT	Depth (m)	Area (m^2^ PE^−1^)	HLR (m^3^ m^−2^ d^−1^)	OLR (g COD m^−2^ d^−1^)	AM	AP	AFR (m^3^ h^−1^)	Effluent DO (mg L^−1^)	TSS (Rem %)	COD (Rem %)	NH_4_^+^-N (Rem %)	TN (Rem %)	TP (Rem %)	Author
HFCW															
Lab	FF	0.3	7	0.03	16.2	CA	B	0.12	NA	95	90	98.5	NA	NA	1
Pilot	LL	0.45	0.8	0.4	357	IA	B	108	NA	NA	60	96	NA	NA	2
Pilot	D	1.0	3.4	0.06	35.3	IA	F	60	0.2–0.6	NA	NA	20 to 89	36 to 86	85 to 85	3
Pilot	S	0.75	8.0	0.1	30.1	IA	B	0.1	3.4	NA	97	95	80	NA	4
Full	D	NA	0.5	0.27	19.4	CA	B	150	8.0–11	69	NA	99	14.1	NA	5
Lab	D	0.38	6.0	0.07	19.7	CA	B	0.5–0.7	6.8	89 to 95	69 to 79	9 to 99	23 to 34	NA	6
Lab	S	0.3	10.8	0.06	11	NA	F	NA	3	NA	90.1	99.7	51.3	NA	7
Lab	S	0.3	10.8	0.06	11	NA	M	NA	NA	NA	76.5	99.7	40	NA	7
Lab	S	0.3	10.8	0.06	11	NA	R	NA	3.2	NA	72.8	99.7	40	NA	7
Lab	D	0.7	11	0.10	11	NA	F	0.24	0.27	NA	82	49	42	NA	8
Pilot	D	1.10	14.1	0.07	8.5	CA	B	12.1	7.0–8.0	NA	64	99	50	NA	9
Pilot	D	1.10	14.1	0.07	8.5	IA	B	12.1	0.5–2.0	NA	54	99	79	NA	9
Lab	P	0.5	3.5	0.03	27	IA	B	0.18	1.9–4.2	NA	64	94	52	NA	10
HCW															
Pilot ^(H + F + H)^	D	1.0	2.3	1.6	51	NA	S	NA	2.04	87	85	81	82	67	11
Pilot ^(H + F + H)^	D	1.0	1.2	3.2	102	NA	S	NA	2.22	89	85	83	83	64	11
Lab ^(H + H)^	D	0.38	6.0	0.07	19.7	CA	B	0.5–0.7	6.8	89 to 91	69 to 82	9 to 57	23 to 41	NA	12
Lab ^(V + H)^	D	0.8/0.35	2.6	0.046	37	IA	B	0.12	2.5	84 to 89	91 to 95	55 to 72	78 to 71	44 to 66	13

The population equivalent (PE) is calculated based on the common relation 1 PE = 60 g BOD d^−1^. Biochemical oxygen demand (BOD) values were approximated using the ratio COD/BOD = 2 in the studies where BOD was not reported (Ouellet-Plamondon et al. 2006; Fan et al. 2013c; Zhong et al. 2015; Zapater-Pereyra et al. 2015; Uggetti et al. 2016). (1) Ouellet-Plamondon et al. (2006), (2) Nivala et al. (2007), (3) Zhang et al. (2010), (4) Fan et al. (2013c), (5) Butterworth et al. (2013), (6) Zapater-Pereyra et al. (2014), (7) Li et al. (2014), (8) Zhong et al. (2015), (9) Uggetti et al. (2016), (10) S. Wu et al. (2016b), (11) Ye and Li (2009), (12) Zapater-Pereyra et al. (2014), (13) Zapater-Pereyra et al. (2015)

*AA* artificial aeration, *HFCW* horizontal flow constructed wetland, *HCW* hybrid constructed wetland, *WT* wastewater type, *FF* fish farm, *LL* landfill leachate, *D* domestic, *S* synthetic, *P* piggery, *HLR* hydraulic loading rate, *OLR* organic loading rate, *AM* aeration mode, *CA* continuous aeration, *IA* intermittent aeration, *AP* aeration position, *B* bottom, *M* middle, *S* surface, *F* front, *R* rear, *AFR* airflow rate, *DO* dissolved oxygen, *TSSs* total suspended solids, *COD* chemical oxygen demand, *NH*_*4*_^*+*^ *-N* ammonium-nitrogen, *TN* total nitrogen, *TP* total phosphorus, *NA* not available, *H + F + H* HFCW free water surface flow CW and HFCW in a stack design, *H + H* HFCW and horizontal flow filter connected in series, *V + H* VFCW over horizontal flow filter

##### Influence of AA on footprint reduction

The footprint of different types of CWs is different with the application of AA (Table 5). For instance, Foladori et al. (2013) investigated the application of AA on pilot-scale VFCW to treat domestic wastewater. The system established simultaneous nitrification and denitrification favorable for high TN removal up to 5.6 g N m^−2^ d^−1^ than the conventional VFCW, which could remove only 1.5 g N m^−2^ d^−1^. The footprint of AA-VFCW was reduced to 1.8 m^2^ PE^−1^, which is half than the footprint (3.6 m^2^ PE^−1^) of the conventional VFCW (Table 5).

Zhang et al. (2010) found in their study on a pilot-scale HFCW, which included aerated and planted, planted, aerated, and control CWs for the treatment of domestic wastewater that due to high removal efficiency (16.7 g BOD m^−2^ d^−1^, 4.54 g NH_4_^+^-N m^−2^ d^−1^, and 4.99 g TN m^−2^ d^−1^), the AA-HFCW requires less footprint of 3.4 m^2^ PE^−1^ (Table 6) compared with traditional HFCW having the footprint of 5 m^2^ PE^−1^ (Vymazal 2011) (Table 1). The removal of NH_4_^+^-N and TN was increased 69 and 50%, respectively, with AA-HFCW. Although the TP removal was not enhanced, it was more stable with aeration as the better mixing with aeration promoted the formation of precipitates (Table 6).

Zapater-Pereyra et al. (2014) studied the effects of AA on the treatment performance of HFCW and HCW, the combination of HFCW and HFF. It was concluded that AA increased the system efficiency per unit area. The AA-HFCW and AA-HCW required 6 m^2^ PE^−1^ footprint, which is less than the control conventional HFCW by 1.9 and 1.5 times, respectively, for COD removal and 49 and 13 times less for NH_4_^+^-N removal (Table 6). Since with AA, an additional removal of COD up to 13% was achieved in HCW, the removal of NH_4_^+^-N was increased 90 and 48% in AA-HFCW and AA-HCW, respectively. Similarly, the TN removal was increased 11 and 18% in AA-HFCW and AA-HCW, respectively (Table 6).

Based on the available evidence, it could be suggested that in all types of CWs, the footprint of the system was reduced with the application of AA. However, AA-HCW and AA-HFCW required small footprint 1.2–6 and 3.4–6 m^2^ PE^−1^ (Table 6) compared with the AA-VFCW, having the footprint of 1.8 to 9.9 m^2^ PE^−1^ (Table 5).

Several other studies also demonstrated the enhanced performance of VFCW, HFCW, and HCW with the application of AA. The influence of AA on organic matter, nitrogen, and phosphorus removal and comparison for the footprint reduction can be seen in Tables 5 and 6.

### Footprint and removal efficiency by aeration method and wetland type

**Fig. 1:**
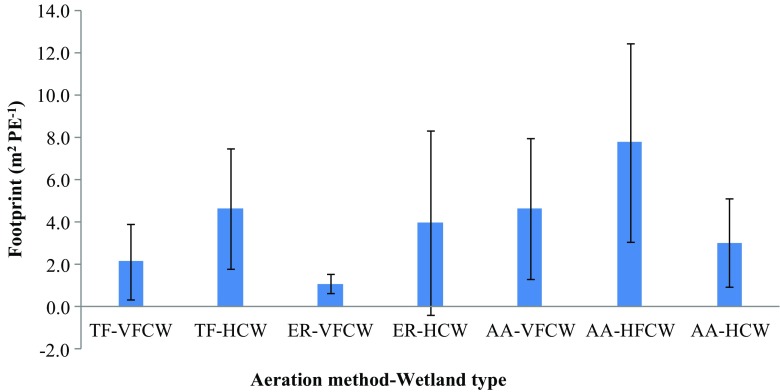
Footprint of the studied aeration methods and wetland types. *Thick and thin bars* represent the mean and standard deviation, respectively. Note that the number of studies is different by aeration method and wetland type; thus, number of data points were 17, 3, 7, 12, 13, 13, and 4 in case of TF-VFCW, TF-HCW, ER-VFCW, ER-HCW, AA-VFCW, AA-HFCW, and AA-HCW, respectively

The footprint of different aeration methods and wetland types, showing the mean and standard deviation estimated from all the studied reviewed in this paper, is summarized in Fig. 1. Notable from this figure is the small footprint of ER-VFCW (1.1 ± 0.5 m^2^ PE^−1^) and TF-VFCW (2.1 ± 1.8 m^2^ PE^−1^). The largest footprint relates to AA-HFCW (7.8 ± 4.7 m^2^ PE^−1^). The footprint of HCW with TF, ER, and AA is 4.6 ± 2.9, 4.0 ± 4.3, and 3.0 ± 2.1 m^2^ PE^−1^, respectively. When footprint is the major indicator for the selection of wetland type, the most obvious choice could be VFCW with most suitable aeration method that could be selected from ER, TF, and AA.

**Fig. 2:**
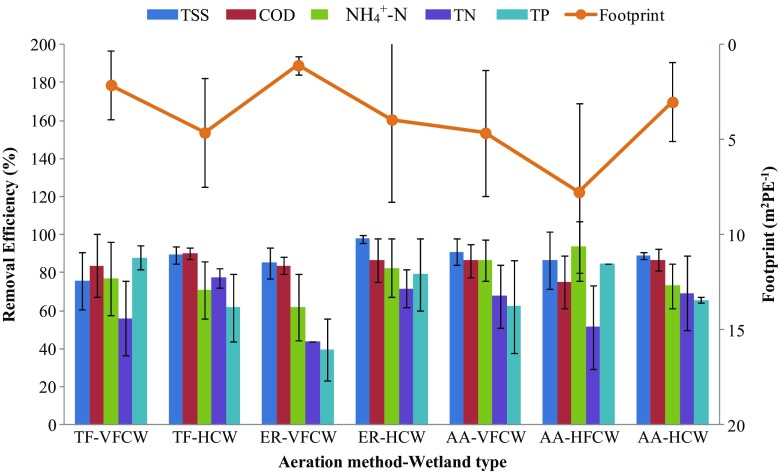
Comparison of removal efficiencies and footprint by aeration method and wetland type. Note that the number of data points used in these calculations was different. TF-VFCW had 6, 15, 17, 15, and 7 observations in case of TSS, COD, NH_4_^+^-N, TN, and TP, respectively. TF-HCW had 3, 3, 3, 3, and 3 observations in case of TSS, COD, NH_4_^+^-N, TN, and TP, respectively. ER-VFCW had 7, 5, 7, 1, and 3 observations in case of TSS, COD, NH_4_^+^-N, TN, and TP, respectively. ER-HCW had 9, 10, 9, 12, and 9 observations in case of TSS, COD, NH_4_^+^-N, TN, and TP, respectively. AA-VFCW had 2, 14, 16, 16, and 9 observations in case of TSS, COD, NH_4_^+^-N, TN, and TP, respectively. AA-HFCW had 3, 11, 13, 11, and 1 observations in case of TSS, COD, NH_4_^+^-N, TN, and TP, respectively. AA-HCW had 4, 4, 4, 4, and 3 observations in case of TSS, COD, NH_4_^+^-N, TN, and TP, respectively

However, removal efficiency is also among the major indicators in the decision process. Therefore, removal efficiencies of the studied aeration methods and wetlands types are summarized in Fig. 2 along with the indicative footprint estimates. The information presented in this figure provides useful reference for the selection of a wetland system for practical application in a given situation.

The highest treatment efficiencies are demonstrated by ER-HCW with removal efficiencies of 98 ± 2, 87 ± 11, 83 ± 15, 72 ± 10, and 79 ± 19 for TSS, COD, NH_4_^+^-N, TN, and TP, respectively. The lowest performance was shown by ER-VFCW with removal efficiencies of 85 ± 8, 84 ± 4, 62 ± 17, 44, and 40 ± 69 for TSS, COD, NH_4_^+^-N, TN, and TP, respectively. Therefore, when footprint and removal efficiencies are equally important, TF-VFCW, AA-HCW, and ER-HCW could be recommended as the best options.

## Conclusion

1. The overall footprint of VFCW, HFCW, and HCW with mean and standard deviation was 2.8 ± 2.7, 8.6 ± 4.7, and 3.9 ± 3.7 m^2^ PE^−1^, respectively, indicating the large footprint by HFCW.
2. Stack design leads to the formation of HCW and contributed in the reduction of footprint with mean and standard deviation of 3.2 ± 1.9 m^2^ PE^−1^.
3. The footprint showed large variation among different types of CWs and aeration methods. ER-VFCW has the small footprint of 1.1 ± 0.5 m^2^ PE^−1^ followed by TF-VFCW with the footprint of 2.1 ± 1.8 m^2^ PE^−1^, and AA-HFCW has the large footprint of 7.8 ± 4.7 m^2^ PE^−1^. The footprint of HCW with TF, ER, and AA is 4.6 ± 2.9, 4.0 ± 4.3, and 3.0 ± 2.1 m^2^ PE^−1^, respectively.
4. Similar to the footprint, a large variation in removal efficiencies is demonstrated by the studied CWs. The synthesis shows that HCW demonstrates highest removal efficiencies followed by the VFCW. The relative contribution of VFCW is much higher than HFCW in overall removal efficiency of HCW. The VFCW contributes more for COD, NH_4_^+^-N, and TP removal, but the HFCW contributes more for TN removal.
5. When footprint and removal efficiency of CWs are equally important then TF-VFCW, AA-HCW and ER-HCW can be considered as the best options.

## Recommendations

**Fig. 3:**
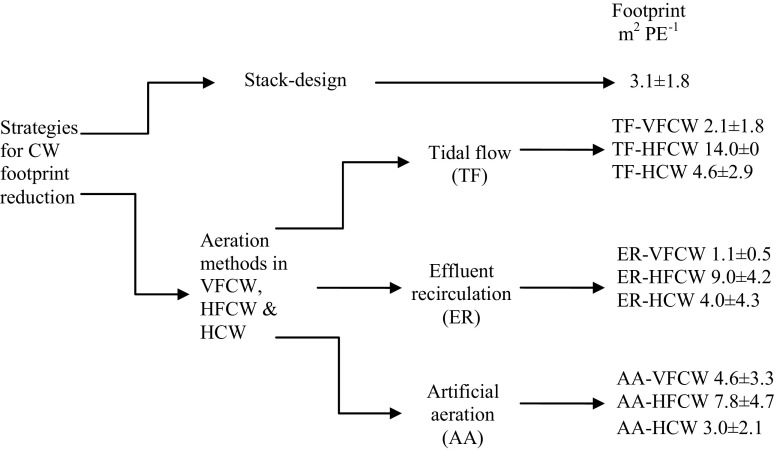
A graphical summary of footprint of different CWs examined in this study

1. Stack design of CWs for the reduction of footprint that is recommended, as the treatment performance can also be enhanced with more than one stage and/or type of CWs.
2. The promising results with TF-VFCW, AA-HCW, and ER-HCW for the treatment performance while reducing the footprint demonstrate high potential for practical applications of these systems.
3. In most of the studies using TF, ER, and AA, the removal of TSS, TN, and TP was not investigated. These parameters need consideration in future research.
4. The data, results, and several new insights presented in this review could be instructive for improved understanding, guiding future studies and practical applications for wastewater treatment. In addition to material presented in this paper, Fig. 3 provides a quick guide of wetland types, intensification strategies, and corresponding footprint, which could be useful for researchers and practitioners.
